# Sublingual nucleotides and immune response to exercise

**DOI:** 10.1186/1550-2783-9-31

**Published:** 2012-07-10

**Authors:** Sergej M Ostojic, Milos Obrenovic

**Affiliations:** 1 Exercise Physiology Laboratory, Center for Health, Exercise and Sport Sciences, Stari DIF, Deligradska 27, Belgrade, 11000, Serbia

**Keywords:** Immunoglobulin A, Exercise, Natural killer cell, Supplementation

## Abstract

Evidence exists regarding the potential role of exogenous nucleotides as regulators of the immune function in physically active humans, yet the potential use of nucleotides has been hindered by their low bioavailability after oral administration. We conducted a double-blind, placebo-controlled, randomized trial to assess the effect of sublingual nucleotides (50 mg/day) on salivary and serum immunity indicators as compared to placebo, both administered to healthy males aged 20 to 25 years for 14 days. Sublingual administration of nucleotides for 14 days increased serum immunoglobulin A, natural killer cells count and cytotoxic activity, and offset the post-exercise drop of salivary immunoglobulins and lactoferrin (P < 0.05), with no adverse effects reported. No significant differences in fasting salivary antimicrobal proteins (α-amylase, lysozyme and lactoferrin) were found before or after the treatment (P > 0.05). It seems that sublingual administration of nucleotides for two weeks considerably affected immune function in healthy males.

## Introduction

Nucleotides are a group of molecules that, when linked together, form the building blocks of RNA and DNA, participate in cellular signaling (e.g. cyclic guanosine and adenosine monophosphates), and are incorporated into important cofactors of enzymatic reactions (e.g., coenzyme A, flavin adenine dinucleotide, flavin mononucleotide, and nicotinamide adenine dinucleotide phosphate). Nucleotides are synthesized endogenously and have important effects on the growth and development of cells with a rapid turnover, such as those of the immune system [1]. However, under certain circumstances exogenous nucleotides may be semi-essential, optimizing the function of the immune system when the endogenous supply may limit the synthesis of nucleotides. Exogenous nucleotides appear to be required for the maintenance of the host immunity in impaired immune responses, such as heavy exercise-related suppression of immune parameters [2]. Oral supplementation with nucleotides in physically active males may offset the hormonal response associated with demanding endurance exercise [3], and boost immune responses to a short term high intensity exercise [4]. Yet, its use is hampered by low bioavailability following oral administration [5]. To avoid the degradation of nucleotides in the gastrointestinal tract and first pass metabolism in the liver after oral intake, sublingual administration of nucleotides may be the more advantageous route of application. No studies so far examined the immunostimulatory effects of sublingual nucleotides in humans. Therefore, we investigated whether daily sublingual administration of 50 mg of nucleotides formulation for 14 days affected indicators of the immune system at baseline and post-exercise in young healthy men.

## Methods

We conducted a double-blind, placebo-controlled, randomized pilot trial to assess the effect of sublingual nucleotides (50 mg daily divided into three portions to be taken at regular intervals throughout the day) as compared to placebo, both administered for 14 days in healthy male participants aged 20 to 25 years. A total of 38 participants were randomly assigned to receive nucleotides (*n* =19) or placebo (*n* =19) and were instrumented for saliva and blood sampling, and endurance running test at the start (day 0) and at the end of the intervention period (day 14). Placebo (inulin) was similar in appearance, volume and taste. The two groups (nucleotides *vs.* placebo) were homogenous for age, height, body mass index, body fat, and maximal oxygen uptake. Venous blood samples were collected after an overnight fast, with white blood cell count (WBC), natural killer cells (NKC) number, NKC cytotoxic activity and serum immunoglobulins (IgA, IgM, IgG) concentration determined. Unstimulated saliva samples were provided at morning and following the endurance running test for determination of salivary immunoglobulins (SIgA, SIgM, SIgG) and antimicrobial proteins (α-amylase, lysozyme, lactoferrin). Exercise test was performed according to the incremental protocol using a treadmill system (Trackmaster TMX425C, Newton, KS, USA). The running protocol consisted of 1-min workloads with participants beginning at a running speed of 8 km/h and increased by 2 km/h for each of subsequent workloads until volitional exhaustion. Duration of the running protocol was identical at day 0 and day 14. Participants were asked to maintain their usual dietary intake and not to change their physical activity patterns during the study. Participants were instructed to report any side-effects of administration (e.g. headache, diarrhea, nausea, weight gain) through an open-ended questionnaire. Two-way analysis of variance (ANOVA) with repeated measures was used to establish if any significant differences existed between subjects’ responses over time of intervention (0 *vs.* 2 weeks). Where significant differences were found, the Tukey test was employed to identify the differences. *P* values of less than 0.05 were considered statistically significant. Effects-sizes in two way ANOVA with replication after two weeks of administration were assessed by Cohen statistics, with *r* > 0.24 indicated medium effect of mixed factors. The data were analyzed using the statistical package *SPSS* 16.0*,* PC program (IBM SPSS Data Collection, New York, NY, USA).

## Results

**Figure 1:**
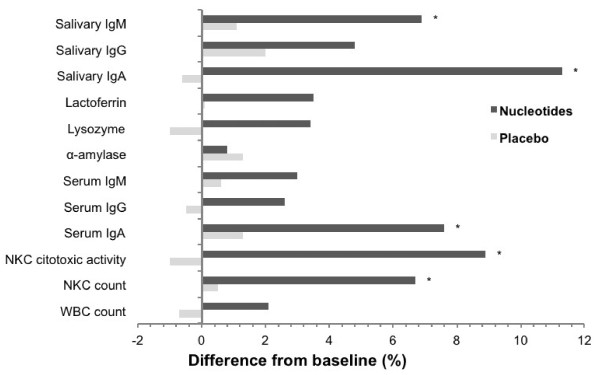
**Percentage change of fasting salivary and serum immunological indices 0*vs.*14 days.** Ig denotes immunoglobulin, NKC natural killer cells, and WBC white blood cells. * Indicates significance (P < 0.05) for the interaction effect (treatment × time).

**Figure 2:**
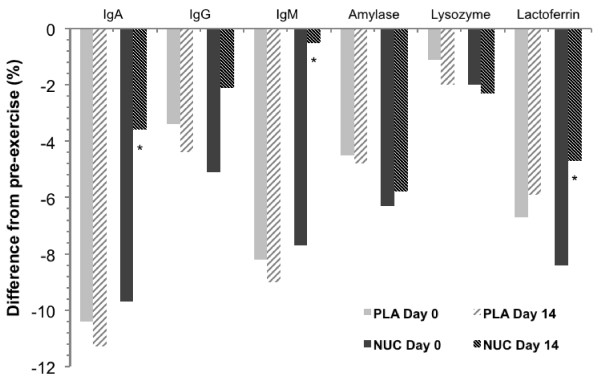
**Post-exercise changes in salivary immunological indices at baseline and after the intervention.** Ig denotes immunoglobulin, PLA placebo group, and NUC nucleotides group. * Indicates significance (P < 0.05) for the pre vs. post administration.

Changes in fasting salivary and serum immunological profiles during the study are presented in Figure 1. Results indicated significant treatment × time interaction for salivary immunoglobulin A (P = 0.0002; *r* = 0.26), salivary immunoglobulin M (P = 0.02; *r* = 0.15), serum immunoglobulin A (P = 0.02; *r* = 0.16), NKC count (P = 0.01; *r* = 0.17), and NKC cytotoxic activity (P = 0.003; *r* = 0.25). Salivary immunoglobulin A increased significantly from before to after administration in nucleotides-administered participants (19.4 ± 3.5 vs. 25.6 ± 5.0 ml/100 mL; 95% CI 3.3–9.1, P < 0.0001; *r* = 0.58). There were no significant differences in salivary and serum immunological outcomes before and after administration in the placebo group. After 14 days of administration, the nucleotides group had higher levels of serum immunoglobulin A than the placebo group (246.8 ± 22.5 vs. 201.4 ± 16.9 μmol/L, 95% confidence interval [CI] 32.3–58.5, P < 0.0001; *r* = 0.75), and higher levels of NKC cytotoxic activity (50.4 ± 14.5 vs. 29.3 ± 8.7 LU, 95% CI 13.2–29.0, P < 0.0001; *r* = 0.66). Salivary measures of immunity were significantly lower after the exercise trial in both nucleotides and placebo groups before as well as after the administration period (P < 0.05). Yet, administration of nucleotides for 14 days significantly diminished the drop of salivary immunoglobulins A (P =0.04; *r* = 0.13), salivary immunoglobulins M (P = 0.004; *r* = 0.18), and salivary lactoferrin after endurance test (P = 0.04, *r* = 0.08) (Figure 2). No volunteers withdrew before the end of the study and no participants reported any vexatious side effect of the supplementation.

## Discussion

The oral application of nucleotides is not a new concept yet only a few human studies evaluated modulation of the immune response mediated by dietary nucleotides. Exogenous nucleotides have been reported beneficial, especially in infants when the nutrition supply was inadequate, since they positively affect NKC activity and production of interleukin-2 [6], plasma levels of immunoglobulin M [7], and antibody response [8]. In two studies by Mc Naughton and co-workers [3,4] the authors reported an increase in the level of salivary immunoglobulin A in a group of physically active males supplemented with nucleotides for 60 days. In the present study, sublingual administration of nucleotides formulation for 14 days increased serum immunoglobulin A, NKC count and cytotoxic activity, and offset the post-exercise drop of salivary immunoglobulins M and A in healthy volunteers, with no adverse effects reported. This implies that nucleotides are absorbed from the mucous membrane under the tongue, enter the circulation and are available for lymphocyte subpopulation activation and proliferation, and modulation of immunoglobulin production. The precise mechanism of the effects of oral nucleotides on cellular immunity is not clear. Gill [1] suggested that the exogenous nucleotides may either affect initial phase of the antigen processing and lymphocyte proliferation, modulate T-helper cell-mediated antibody production, or mediate signal membrane transduction and expression of a number of genes, some of which can directly affect the levels of cell-signaling protein molecules. Further studies are needed to explicate the mechanism of immunostimulatory effects of the sublingual nucleotides, with longer administration protocol and a higher dosage of the formulation, along with proven bioavailability coupled with monitoring of the other indicators of immunity. Our study suggests that the immunostimulatory potential of sublingual nucleotides in healthy subjects is superior as compared to oral intervention, since oral nucleotides significantly raised salivary immunoglobulin A by up to 5% and attenuate the drop in post-exercise IgA by up to 3% [3], while bioavailability after oral nucleotides administration was less than 10% [5,9].

In conclusion, it appears that sublingual nucleotides (50 mg per day) strongly influence immunity in healthy males when administered for two weeks, with a significant increase in fasting salivary and serum immunity indicators. Participants supplemented with nucleotides experienced reduced post-exercise drop of salivary immunoglobulins M and A for up to 7%. Salivary nucleotide supplement had an acceptable safety profile with no incidence of side-effects reported.

## Abbreviations

WBC, White blood cell count; NKC, Natural killer cells; Ig, Immunoglobulin; CI, Confidence interval.

## Competing interest

The author(s) declare that they have no competing interests.

## Authors' contributions

SMO was responsible for the study design, biochemical work, statistical analyses, and manuscript preparation. MO was responsible for literature review and manuscript preparation. Both authors read and approved of the final manuscript.
